# The Asylum Journal
*The Asylum Journal. (Thirteen Numbers published.) Edited by Dr. Bucknill, Resident Physician of the Devon County Lunatic Asylum. Highley, Fleet-street. Monthly, price 6d.


**Published:** 1855-01-01

**Authors:** 


### Art. V.—THE ASYLUM JOURNAL.*

This journal was projected at a meeting of the "Association of Medical Officers of Hospitals and Asylums for the Insane," held at Oxford in the month of July, 1852. Some gentlemen connected with the management of public asylums thought it desirable to publish an occasional fly-sheet or "Asylum Journal," to circulate principally among the resident medical superintendents of these establishments, in order to afford to these gentlemen a vehicle in which they could compare notes respecting points of practical interest connected with the conduct of such institutions, and the treatment of the insane confided to their professional care. The originators of this publication never contemplated establishing a journal in rivalship with our own. This idea was fairly repudiated at the meeting. It certainly occurred to us when the proposals for the establishment of this new journal were submitted to the consideration of the Oxford meeting, that if any of the medical men connected officially with public asylums had been anxious to bring any point of practical interest before their fellow-labourers or the profession generally, *our* pages would at all times have been open to them; but as they had not thought proper to send their communications to the *Psychological Journal*, we were at the time doubtful whether a periodical of less pretensions would receive such an amount of literary support as to render it at all useful to those for whose special perusal it was intended.

Dr. Bucknill, the well-known and intelligent physician of the Devon County Asylum, was unanimously selected to conduct the journal for the Association. It is now our pleasing duty to direct the attention of our readers to the result of his editorial labours. Dr. Bucknill has, we have no doubt, found, ere this, that in editing a periodical of this kind, he must, in the main, rely upon his own exertions. He has un-

* The Asylum Journal. (Thirteen Numbers published.) Edited by Dr. Bucknill, Resident Physician of the Devon County Lunatic Asylum. Highley, Fleet-street. Monthly, price 6d.

doubtedly had afforded him some degree of literary assistance, but not, we dare say, to the extent he anticipated when he consented to mount the editorial chair. Having been a few years in advance of Dr. Bucknill in wielding the editorial baton, we can speak practically of the difficulties that have beset our path in the conduct of a periodical like our own, devoted to the discussion of medico-psychological literature. We do not complain of the obstacles that have occasionally interfered with our successful onward march, or look back upon the past with any feelings of pain or regret. Our labours have been those of love. We were disposed at one time to entertain the opinion that some of the "veterans" connected with the department of the science of medicine we were engaged in cultivating, might have extended to us *some* degree of literary assistance, and occasionally have held out to us a helping-hand; but they, with a few brilliant exceptions, neither did one nor the other, from motives best known to themselves. Dr. Bucknill has, we have no doubt, experienced, in the editorship of his unassuming journal, difficulties somewhat analogous to those which, in a slight degree, somewhat damped our ambition during the earlier periods of the history of the *Psychological Journal;* but, like ourselves, he has triumphed over them, and has fairly launched his fragile bark upon those stormy seas, said to be—

"Bankrupt of life and prodigal of care."

The numbers of the journal before us contain papers of deep interest and great practical importance. The editor has, of course, contributed largely to the pages of the *Asylum Journal.* We refer particularly to a valuable, well-written and practical article on "Bed sores in the Insane," and on the "Head-dress of Pauper Lunatic Men," and to various leading introductory papers that have appeared, from time to time, on subjects of immediate interest. Dr. Arlidge has published in the journal a series of papers "On the Examination of the Brain after Death," well worthy of the student's attentive reading. We all know how loosely, slovenly and carelessly the brain is often examined after death. It therefore behoves all occupied in these delicate and important investigations, to consider well the rules laid down by this physician for the guidance of those engaged in the study of cerebral pathology.

Dr. Boyd's communication on "Cholera" is valuable; but is it not too elaborate for a special periodical of this character? The essay occupies the greater part of one number of the journal. Would it not be better for the future to avoid publishing papers of such length, unless directly bearing upon some point of practical value relative to the organization of asylums or treatment of insanity? The short notices of new publications are characterized by a liberal spirit of criticism, and the works selected for review are, with one exception, chosen with judgment and good taste. The exception to which we refer is a work on "Nervous Diseases," with the name of Dr. Maddock on its title-page. An *ad captandum* publication like this is unworthy of notice in the columns of a scientific journal. The book is evidently written for commercial purposes, and does not contain one point that should redeem it from the hands of the butter-merchant or trunk-maker. When we have so much to commend in Dr. Bucknill's editorial management we regret to be obliged, from a sense of duty and justice, to view any matter in a light different to that in which he has himself discussed it. In Nos. 6 and 7 of the journal Dr. Bucknill has considered it necessary to animadvert strongly on Dr. Simpson's management of the Lunatic Asylum for the North and East Riding of Yorkshire, in reference to the use of the milder forms of mechanical restraint under special and pressing circumstances.

The Commissioners in Lunacy, in their official entry made after a careful inspection of this public asylum on the 18th of March, 1854, refer to four cases, in the treatment of which Dr. Simpson conceived it to be his duty to apply, temporarily, mechanical restraint. In a subsequent part of their report they observe—

"No material alteration has taken place in the general arrangement of the institution since the visit of the Commissioners in June last; but the whole establishment is now on so steady and satisfactory a footing, that the details of its daily management are carried on with great ease and regularity, and we did not observe anything as to which we could suggest any change likely to be useful."

Now, Dr. Bucknill takes grave exception to this laudatory paragraph, and cannot conceive how the Commissioners can say, that they "did not observe anything as to which we can suggest any change likely to be useful," when one of the female patients had, at the time of their visitation, in consequence of her extreme violence, "her hands tied behind her by means of a pocket-handkerchief, and, in three other cases, the spencer had been occasionally employed to prevent acts of violence and destruction." It is not our intention to go in detail into the merits of the matter in dispute between Dr. Simpson and Dr. Bucknill. Dr. B. does not think this establishment can justly be held up as a "pattern institution," or be entitled to the eulogy of the Commissioners, as long as the patients are subjected to any amount of mechanical restraint. Dr. Simpson's very temperate reply to Dr. Bucknill's editorial criticism is published in No. 7 of the *Asylum Journal*, and is well worthy of perusal. Ranking, as we do, among the moderate advocates of a partial use of mechanical restraint, Dr. Bucknill cannot expect that we should take *his* view of the matter in dispute. We can conceive an institution for the treatment of the insane to be a "pattern one," and fully entitled to eulogia similar to that bestowed upon Dr. Simpson by the Commissioners, in which mechanical restraint and seclusion are "occasionally" used for the treatment, safety, and care of its unhappy inmates.

With the exceptions previously referred to, it affords us, personally, much gratification to speak in the warmest terms in praise of Dr. Bucknill's editorial exertions. We can appreciate the difficulties with which he has had to contend, and are ready to make great and liberal allowances for any slight deviations from what we conceive to be the boundaries of legitimate criticism. We propose, from time to time, to bring this journal under the notice of our readers, and when we have more space at command, to quote extensively from its columns.

